# Nano-Structured Lipid Carrier-Based Oral Glutathione Formulation Mediates Renoprotection against Cyclophosphamide-Induced Nephrotoxicity, and Improves Oral Bioavailability of Glutathione Confirmed through RP-HPLC Micellar Liquid Chromatography

**DOI:** 10.3390/molecules26247491

**Published:** 2021-12-10

**Authors:** Adel M. Ahmad, Hamdoon A. Mohammed, Tarek M. Faris, Abeer S. Hassan, Hebatallah B. Mohamed, Mahmoud I. El Dosoky, Esam M. Aboubakr

**Affiliations:** 1 Department of Pharmaceutical Analytical Chemistry, Faculty of Pharmacy, South Valley University, Qena 83523, Egypt; 2 Department of Medicinal Chemistry and Pharmacognosy, College of Pharmacy, Qassim University, Buraydah 51452, Saudi Arabia; 3 Department of Pharmacognosy, Faculty of Pharmacy, Al-Azhar University, Cairo 11371, Egypt; 4 Department of Pharmaceutics and Industrial Pharmacy, College of Pharmacy, Al-Azhar University, Cairo 11371, Egypt; tarek_faris05@yahoo.com; 5 Department of Pharmaceutics, Faculty of Pharmacy, South Valley University, Qena 83523, Egypt; abeer.saad@svu.edu.eg (A.S.H.); dochoba2014@svu.edu.eg (H.B.M.); 6 Department of Pathology, Faculty of Medicine, South Valley University, Qena 83523, Egypt; mahmouddosoky@med.svu.edu.eg; 7 Department of Pharmacology and Toxicology, Faculty of Pharmacy, South Valley University, Qena 83523, Egypt; esam_pharma@yahoo.com

**Keywords:** cyclophosphamide, anticancer, acrolein, renal toxicity, glutathione, nanostructured lipid carrier, GSH-NLCs, micellar liquid chromatography, RP-HPLC

## Abstract

The study aimed to develop a new glutathione (GSH) oral formulation to enhance the delivery of GSH and counter the nephrotoxicity of the anticancer drug, cyclophosphamide (CP). A nanostructured lipid carrier glutathione formulation (GSH-NLCs) composed of glutathione (500 mg), stearic and oleic acid (300 mg, each), and Tween^®^ 80 (2%, *w*/*v*) was prepared through the emulsification-solvent-evaporation technique, which exhibited a 452.4 ± 33.19 nm spheroidal-sized particulate material with narrow particle size distributions, −38.5 ± 1.4 mV zeta potential, and an entrapment efficiency of 79.8 ± 1.9%. The GSH formulation was orally delivered, and biologically tested to ameliorate the CP-induced renal toxicity in a rat model. Detailed renal morphology, before and after the GSH-NLCs administration, including the histopathological examinations, confirmed the ameliorating effects of the prepared glutathione formulation together with its safe oral delivery. CP-induced oxidative stress, superoxide dismutase depletion, elevation of malondialdehyde levels, depletion of Bcl-2 concentration levels, and upregulated NF-KB levels were observed and were controlled within the recommended and near normal/control levels. Additionally, the inflammatory mediator marker, IL-1β, serum levels were marginally normalized by delivery of the GHS-NLCs formulation. Oral administration of the pure glutathione did not exhibit any ameliorating effects on the renal tissues, which suggested that the pure glutathione is reactive and is chemically transformed during the oral delivery, which affected its pharmacological action at the renal site. The protective effects of the GSH-NLCs formulation through its antioxidant and anti-inflammatory effects suggested its prominent role in containing CP-induced renal toxicity and renal tissue damage, together with the possibility of administrating higher doses of the anticancer drug, cyclophosphamide, to achieve higher and effective anticancer action in combination with the GSH-NLCs formulation.

## 1. Introduction

The spontaneous uncontrolled proliferation of cells, named cancers, represents complicated disorders, and one of the most common diseases that affects a large population in the world. Moreover, cancer threatens the lives of millions around the world and consequently affects the quality of life and life duration of cancer patients [1,2]. In 2019, the World Health Organization (WHO) identified cancer as one of the leading causes of death globally [3]. Therefore, studies on drug discovery and delivery for cancer treatment are of global interest and their progression has increased dramatically in recent years in order to find new anticancer candidates, improve drug selectivity, and reduce the side effects of current anticancer drugs, which are the major challenges in cancer drug therapy [4,5,6]. Common side effects have been recorded for chemotherapeutic agents. Some of these side effects were described as critical, e.g., severe vomiting, diarrhea, myelosuppression, and liver and renal impairments [7]. However, continuous trials have attempted to provide alternative dosage forms for chemotherapeutic agents and co-administration with other curative drugs to solve this problem [8,9,10,11].

Cyclophosphamide (CP) is one of the most effective chemotherapeutic agents, used primarily for cancer management and as immunosuppressive therapy [12,13]. It is used in chemotherapy protocols for a wide range of malignancies, including carcinomas, sarcomas, feline lympho-proliferative disorders, mast cell tumors, breast carcinomas, and lymphoma [14,15,16]. Unfortunately, CP-induced acute inflammation of the urinary bladder (cystitis, hemorrhagic cystitis) and renal and liver damage hinder its utilization in the treatment of many cancer types [17,18].

The nephrotoxicity of CP is mostly attributed to its toxic metabolites, i.e., acrolein and phosphoramide [19,20]. The acrolein toxicity is attributed to oxidative stress and the formation of adduct with the renal cellular glutathione (GSH), leading to its cellular depletion [18,21,22]. GSH cellular depletion limits its biological function in the GSH-dependent antioxidant system, leading to an increase of free radicals inside nephrons, and resulting in necrosis of tubular epithelial cells [21,22,23]. On the other hand, oral administration of GSH is not recommended as an option to reduce nephrotoxicity of CP, since oral GSH intake is rapidly degraded by gamma-glutamyl transpeptidase enzyme, thus losing its functions [24].

Various nanoparticles preparations have been used to improve drug delivery and the therapeutic index of anticancer active natural and synthetic products [6,25]. Lipid-based nano- and microparticles, such as solid lipid nanoparticles (SLNs), solid lipid microparticles (SLMs), and nanostructured lipid carriers (NLCs), have been reported as efficient carriers for lipophilic drugs [26]. The mixture of liquid oils and solid lipids in the formulation of NLCs results in the formation of less ordered lipid matrixes, which enhance the drug loading capacity and stability. Liquid lipid’s role in NLCs is to distort the lipid crystals’ formations, which enhances the stability of the prepared nanoparticles during storage, reducing drug leakage, and particle gelation. Liquid lipids also improve the drug loading efficiency and decrease the particle size of the prepared nanoformulation [27]. Furthermore, NLCs have been invented primarily to withstand storage and overcome drug expulsion from SLNs due to crystallization and phase transition of the solid lipids [28].

This study aimed to evaluate the bioavailability and renal protection efficacy of GSH loaded as NLCs. GSH-NLCs were formulated using the emulsification-solvent evaporation technique followed by ultrasonication. The physicochemical characteristics, i.e., particle size, zeta potential, encapsulation, and loading efficiency, of the GSH-NLCs were evaluated. Furthermore, the protective effects of GSH-NLCs against CP-induced nephrotoxicity, compared to pure GSH, were investigated as part of the current research. The GSH concentrations in the renal tissue homogenates of injured and treated animals were also measured using a new validated micellar liquid chromatographic analysis method. The intracellular redox status and oxidative stress were assessed by determining the levels of malondialdehyde, reduced GSH, and superoxide dismutase in renal tissue. The inflammatory state was determined by assessing the serum levels of IL-1β, and both the NF-KB and Bcl-2 concentrations in the renal tissue. On the other hand, kidney functions were evaluated by determining urea and creatinine serum concentrations. Additionally, renal tissue damage was assessed using histopathological examination in an attempt to validate the effects of the GSH-NLCs on the cyclophosphamide-induced renal tissue damage.

## 2. Results

### 2.1. Preparation and Characterizations of GSH-Loaded Nanostructured Lipid Carriers

Nanostructured lipid carriers with GSH concentrations of 50 mg/mL were successfully prepared using emulsification-solvent evaporation technique followed by ultrasonication. The oleic acid (liquid lipid) and stearic acid (solid lipid) were selected based on the preliminary study for glutathione solubility and entrapment in these lipids. The effect of different ratios of solid lipid and liquid lipid on the glutathione encapsulation into NLCs was investigated (Table 1). It was noticed that the higher solid lipid to liquid lipid ratios (F7 contains 300 mg of oleic acid and 300 mg of stearic acid) formed highly stable nanostructures with improved encapsulation efficiency (EE value of 79.8 ± 1.9%). On the other hand, the hydrophilic surfactant, Tween^®^ 80, has a high value of HLB (hydrophilic-lipophilic balance) that governs the effectiveness of the emulsification process and stabilization of the oil nanodroplets during the solvent evaporation process. Characterization of the developed formulation was carried out by measuring the particle size, PDI, zeta potential, and loading and encapsulation efficiency. Results obtained from Malvern Zeta Sizer showed that the average particle size, PDI, and zeta potential were 452.4 ± 33.19 nm, 0.500 ± 0.12, and−38.5 ± 1.4, respectively. The small particle sizes and higher negative zeta potential value of the NLCs formulation confirmed the stability of the colloidal dispersion. Furthermore, the drug loading and encapsulation efficiency of the GSH-NLCs were found to be 6.78 ± 0.05% and 79.8 ± 1.9%, respectively (Table 2).

#### 2.1.1. Particle Morphology

**Figure 1:**
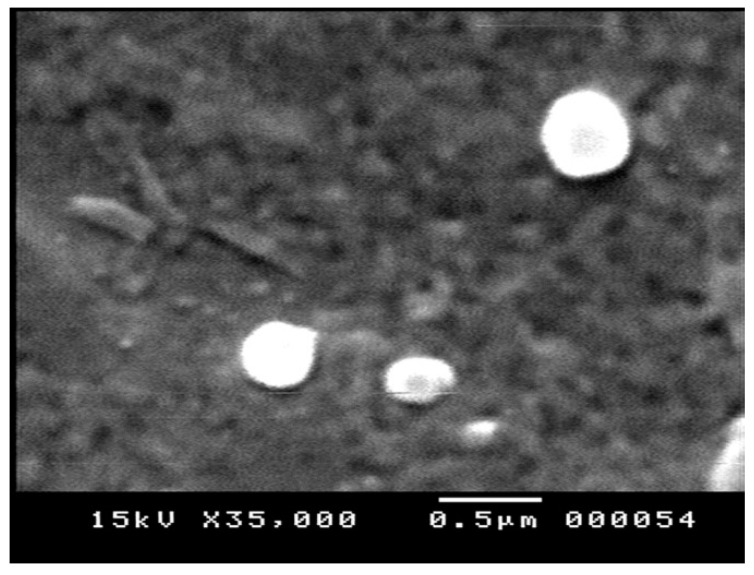
Scanning electron micrograph of the GSH-NLCs.

SEM micrographs (Figure 1) revealed that GSH-NLCs displayed a uniform and spherical shape with no evident sign of aggregation.

#### 2.1.2. In Vitro Release Profile of the Glutathione from NLC Formulation

**Figure 2:**
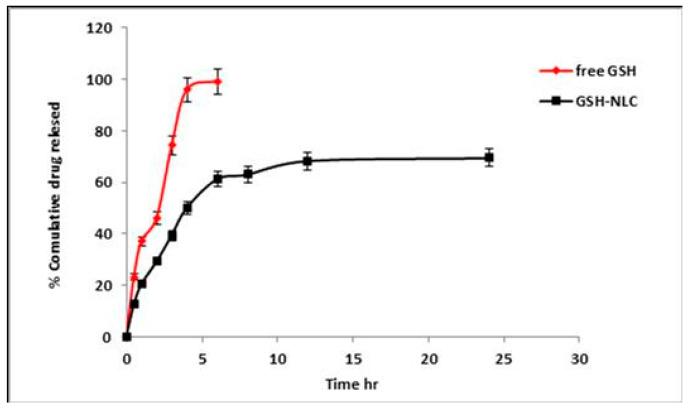
In vitro release profile of the GSH-NLCs formulation compared with free GSH solution in phosphate buffer saline (PBS, pH 7.4 at 37 °C).

The in vitro studies of the GSH-NLCs formulations were performed in PBS, and pH 7.4, at 37 °C, using the modified dialysis membrane diffusion technique. The in vitro release profile of GSH-NLCs was compared with that of the free and pure GSH solution (Figure 2). The GSH solution showed maximum drug release (100% ± 1.00) in 4 h due to the higher water solubility of GSH. The GSH-NLCs formulation demonstrated a biphasic release profile with an initial rapid drug release of 39.42 ± 0.98% in the first 3 h followed by sustained drug release (73.4 ± 1.5%) up to 24 h. The drug release best fit within the Higuchi diffusion model, with a correlation coefficient (r^2^) equal to 0.918.

#### 2.1.3. Fourier Transform-Infrared (FT-IR) Spectroscopy

**Figure 3:**
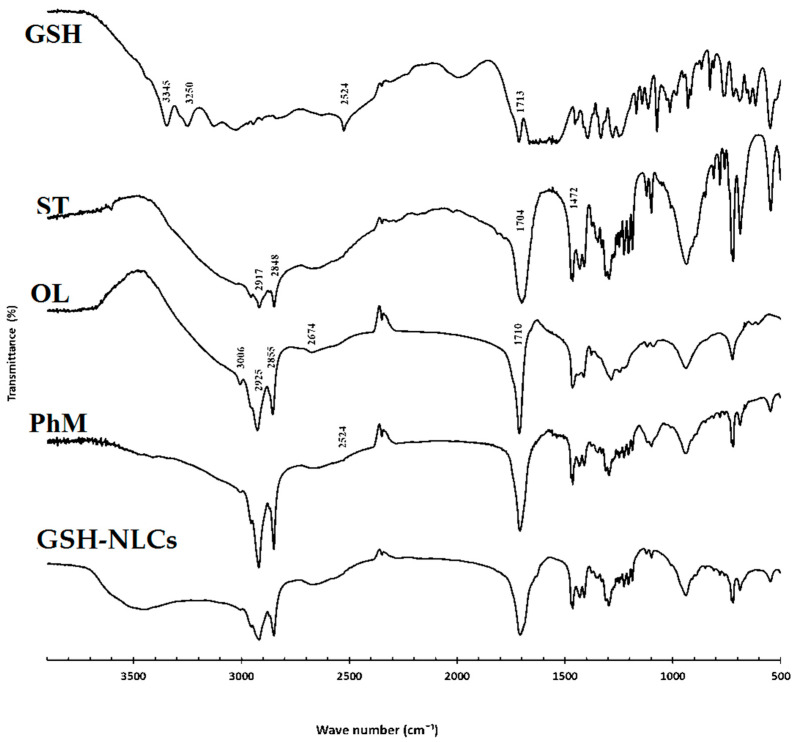
FT-IR spectra of the pure drug (GSH), stearic acid (ST), oleic acid (OL), physical mixture (PhM), and glutathione nanostructure lipid carriers (GSH-NLCs).

FT-IR analyses were performed in the range of 4000–400 cm^−1^ to detect any chemical or physical interactions between glutathione and other components in the physical mixture and the NLCs (spectra of the GSH, stearic acid, oleic acid, and GSH-NLCs are displayed in Figure 3). The diagnostic peaks of GSH were found at 2524, 3445, 3250, and 1713 cm^−1^ (S-H stretching, N-H stretching, and C=O stretching vibration, respectively) in the GSH spectrum [29]. Further, the diagnostic peaks of stearic acid for the aliphatic C–H (2917 and 2849 cm^−1^), C=O (1704 cm^−1^), and C–O (1472 cm^−1^) vibrations were shown in the spectrum of the compound [30]. It was also noticed that the distinctive peak of the GSH at 2524 cm^−1^ (S-H stretching vibration) was detected in the FT-IR spectrum of the NLCs physical mixture (very small peak), which might indicate that no chemical interaction between GSH and other components of the formulation took place. The peaks for stearic and oleic acid were observed, which confirmed the drug was encapsulated inside the provided lipid matrix.

### 2.2. Stability Studies

The GSH-NLCs’ long-term stability was investigated at 4 and 25 °C (room temperature) for 30 days. Visual examinations of the GSH-NLCs, stored for one month at both temperatures, i.e., 4 °C and RT, demonstrated no signs of gelation, creaming, or particle aggregation in the formulation. In addition, the particle size and zeta potential values of the GSH-NLCs were not significantly different after one month of storage (Table 3). The size was 580.2 and 598.9 nm at 4 °C and room temperature, respectively, with PDI near to 0.5. The zeta potential was close to −30 mV for the 30 days of storage, and a −2.0 reduction in the zeta potential was observed. A seepage of about a 2.5% reduction in EE% was observed. Thus, the developed formulation was able to protect the incorporated glutathione from degradation. This represented an important observation, considering that, in many cases, chemical and physical instabilities have been observed when NLCs were stored as an aqueous suspension for long periods, i.e., 30 days and up. This is understandably observed due to lipid crystallization, polymorphic transformations, aggregation phenomena, and hydrolysis processes in the stored formulation.

### 2.3. HPLC Analyses and Validation

Micellar liquid chromatography (MLC) has been established as a versatile analytical tool for a variety of applications. The advantage of MLC over other chromatographic techniques is that physiological fluids can be directly injected into the HPLC, as there is no need to inject high-purity samples. Hydrophilic drugs, such as GSH, usually not retained in RP-HPLC, are often retained in the MLC owing to ionic interactions with the charged surfactant molecules [31].

**Figure 4:**
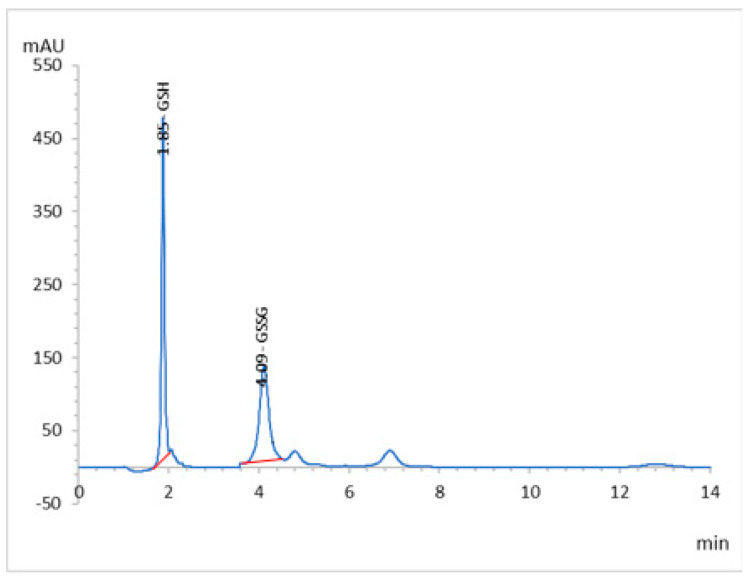
RP-HPLC chromatogram of the GSH and GSSG of working standard mixture (5.0 µgmL^−1^) taken under optimized conditions.

MLC has been applied effectively to analyze GSH in renal tissue homogenates without relentless division. This is because micelles can competitively bind proteins, and release protein-bound drugs and the proteins without sedimentation on the column. In this way, the analysis is performed directly with no requirement for utilization of toxic or flammable organic solvents, which reduces the cost and analysis time, and the separation efficiency is improved. In this work, the retention and selectivity of GSH were affected by different factors, such as the pH of the mobile phase, temperature of the column, percentage of acetonitrile, and concentration of sodium lauryl sulfate (SDS). After optimization, the optimum mobile phase was found to be 95% potassium phosphate buffer (20 mM, pH 2.5) containing 0.15 M SDS and 10% acetonitrile, and the column temperature was set at 30 °C with a mobile phase flow rate of 0.7 mL/min, which showed the retention time at 1.85 min for GSH and 4.09 min for GSSG, the oxidized version of the GSH (Figure 4) at 210 nm. The analytical validation parameters, such as the linearity, accuracy, precision, LOD, and LOQ, are summarized in Table 4 and Table S1 (Supplementary Materials)**.** The system suitability parameters, i.e., tailing factor, number of theoretical plates (TPs), and the height (HTP) equivalent to a theoretical plate (HETP), were calculated, and are summarized in Table 5.

The increase in SDS concentrations showed a sharper decrease in the retention of GSH, and this can be explained by assuming that GSH can interact electrostatically, as well as a hydrophobic nature material with the micelle and stationary phase. The acidic pH of the mobile phase was used to prevent oxidation of the GSH and to suppress the ionization of residual silanols or other active sites on the stationary phases. Retention of GSH in the column decreased due to an increase in the proportion of acetonitrile in the mobile phase because the equilibrium of the solute in the stationary phase and micelles were displaced by the bulk aqueous phase, leading to a reduction of retention factors. The chromatograph showed a retention time of 1.86 min at 210 nm for GSH. A high linearity range (5–25 μg/mL) was shown at an LOD and LOQ value of 0.439 and 1.33 μg/mL, respectively, indicating that the developed method has good sensitivity. The method showed high recovery (99.25 ± 0.88%) and an RSD percentage value less than two, which are under the accepted criteria for this study. Additionally, the precision study showed that the method was found to be precise and accurate.

The tailing factor and asymmetry factor of the 5 µg/mL for the peaks were 1.23 and 0.933, respectively. The theoretical plate numbers were determined to be greater than 2000, and the HEPT was 0.00383 cm. All of the above values were within the acceptable limits and established the system’s suitability for the proposed analysis (Table 5). The well-shaped peaks in the chromatograms verified that the proposed method has satisfactory specificity.

### 2.4. Histopathological Examinations

**Figure 5:**
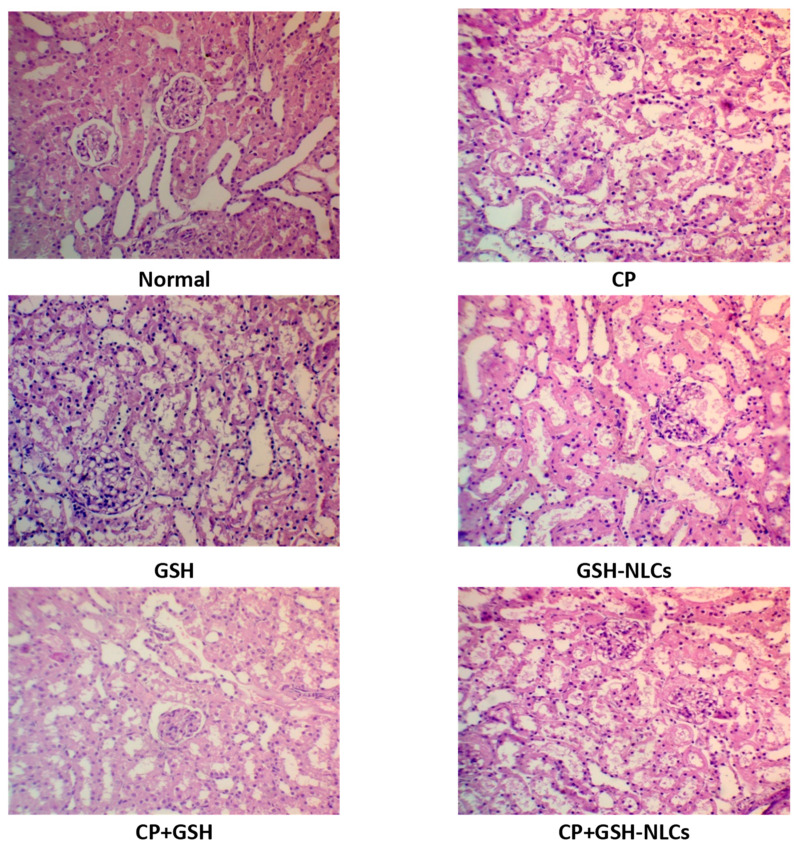
Histopathology of rats’ kidney (H & E stain, magnification ×200) of **Normal** = Normal rats; **CP** = Cyclophosphamide-injured rats; **GSH** = glutathione-treated rats, **GSH-NLCs** = nano-glutathione-treated rats; **CP+GSH** = Cyclophosphamide + Glutathione-treated rats; **CP+GSH-NLCs** = Cyclophosphamide + nano-glutathione-treated rats.

Kidneys of the untreated group showed a normal histological structure, while the CP group examination revealed remarkable tubular inflammatory cell infiltration with degeneration, congestion, and edema, which were associated with the swelling of the endothelial cells that line the glomeruli. On the other hand, oral GSH administration could not produce any significant amelioration of the histopathological changes that were induced by CP. However, GSH-NLCs administration significantly protected renal tissues from damage that was induced by CP, as shown by moderate tubular inflammatory cell infiltration and swelling, which was also accompanied by scanty necrotic tissue (Figure 5).

### 2.5. Renal Functions Tests

**Figure 6:**
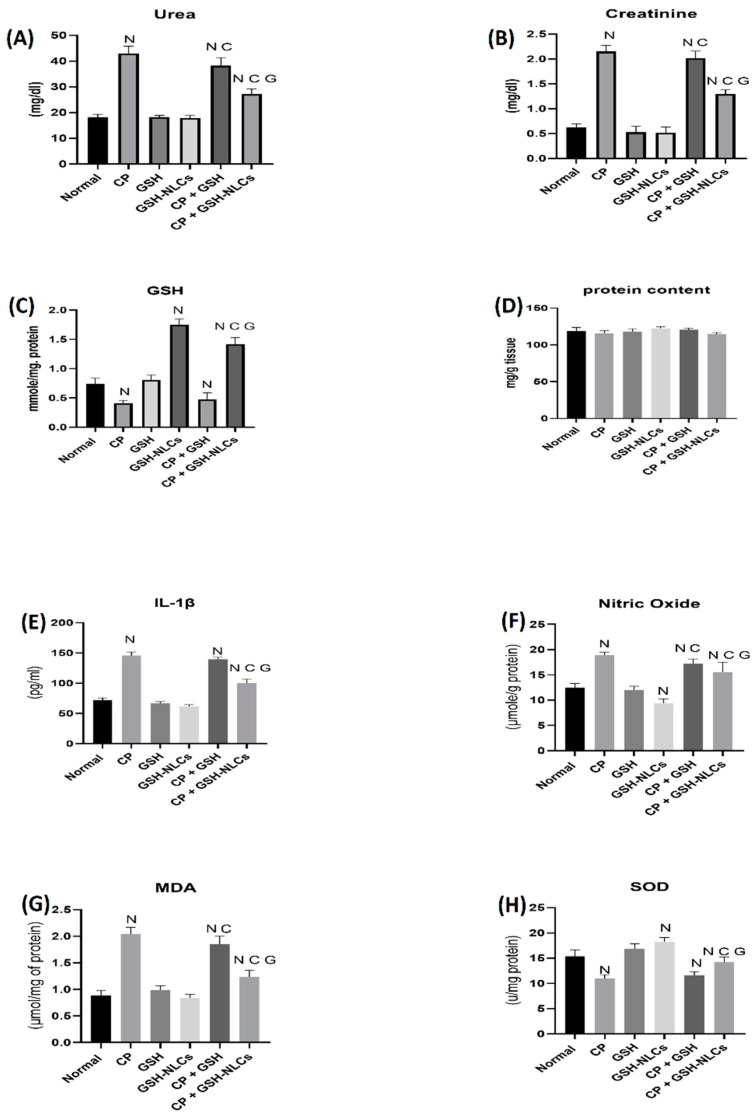
Variations in the levels of biochemical parameters in the normal, injured, and treated animal groups whereas (**A**) urea, (**B**) creatinine, (**C**) GSH, (**D**) protein, (**E**) IL-Iβ, (**F**) nitric oxide, (**G**) MDA, and (**H**) SOD levels in different groups. (N: significantly different than normal; C: significantly different than CP; G: significantly different than the CP+GSH group).

As demonstrated in Figure 6, the urea and creatinine concentrations in the normal group were 18.1 and 0.62 mg/dL, respectively, while the CP administration significantly increased their concentrations up to 43 and 2.1 mg/dL (*p* < 0.05). The oral administration of GSH did not produce significant changes in the CP effects, while GSH-NLCs significantly ameliorated the CP effects, with urea and creatinine concentrations of 27.2 and 1.2 mg/dL, respectively (*p* < 0.05).

### 2.6. Effects on Nitric Oxide

Figure 6 also shows the nitric oxide in the normal group of 12.5 μmol/g level of protein, while CP administration increased its concentration up to 18.8 μmol/g of protein level (*p* < 0.05). However, GSH-NLCs administration significantly inhibited the CP-inducible effect, and the NO concentration was measured at 15.5 μmol/g protein, which was also significantly less than CP+GSH-administered group (*p* < 0.05).

### 2.7. Effects on GSH Concentrations

A reduced glutathione concentration in the normal group of animals was measured by the RP-HPLC method of 0.73 µmol/mg protein (Figure 6). However, this concentration was significantly decreased to 0.41 µmol/mg protein by the CP administration (*p* < 0.05). Nevertheless, the oral administration of GSH-NLCs to the normal non-treated animals group significantly increased the GSH concentration up to 1.7 µmol/mg protein, although GSH-NLCs administration significantly prevented the CP-reducing effects on the GSH, and the GSH concentration was found to be 1.4 µmol/mg protein (*p* < 0.05).

### 2.8. Effects on IL-1β

CP administration significantly increased the IL-1β concentrations compared to the normal group (*p* < 0.05). The co-administration of GSH-NLCs with CP significantly reduced IL-1β (*p* < 0.05), whereas the oral administration of GSH did not affect this CP proinflammatory actions (Figure 6).

### 2.9. Effects on MDA

CP also significantly increased the MDA concentration (Figure 6), as compared to the normal group, up to 2.01 μmol/mg of protein (*p* < 0.05), while this effect was significantly decreased by the CP+GSH co-administration (1.85 μmol/mg protein) (*p* < 0.05). However, GSH-NLCs administration nearly normalized the MDA concentration, and significantly ameliorated the CP effects with an MDA concentration of 1.23 μmol/mg protein (*p* < 0.05).

### 2.10. Effects on SOD

During the present study, the SOD concentration in the normal animal group was found to be 15.3 u/mg protein, while the CP group showed a significant reduction in SOD concentrations (11 u/mg protein, *p* < 0.05). However, GSH-NLCs, when orally administered, remarkably inhibited the CP-declining effects, and the SOD concentration was found to be 14.25 u/mg protein. On the other hand, GSH oral administration did not affected the CP-depleting effect on SOD (Figure 6).

### 2.11. Effects on Bcl-2 Protein

**Figure 7:**
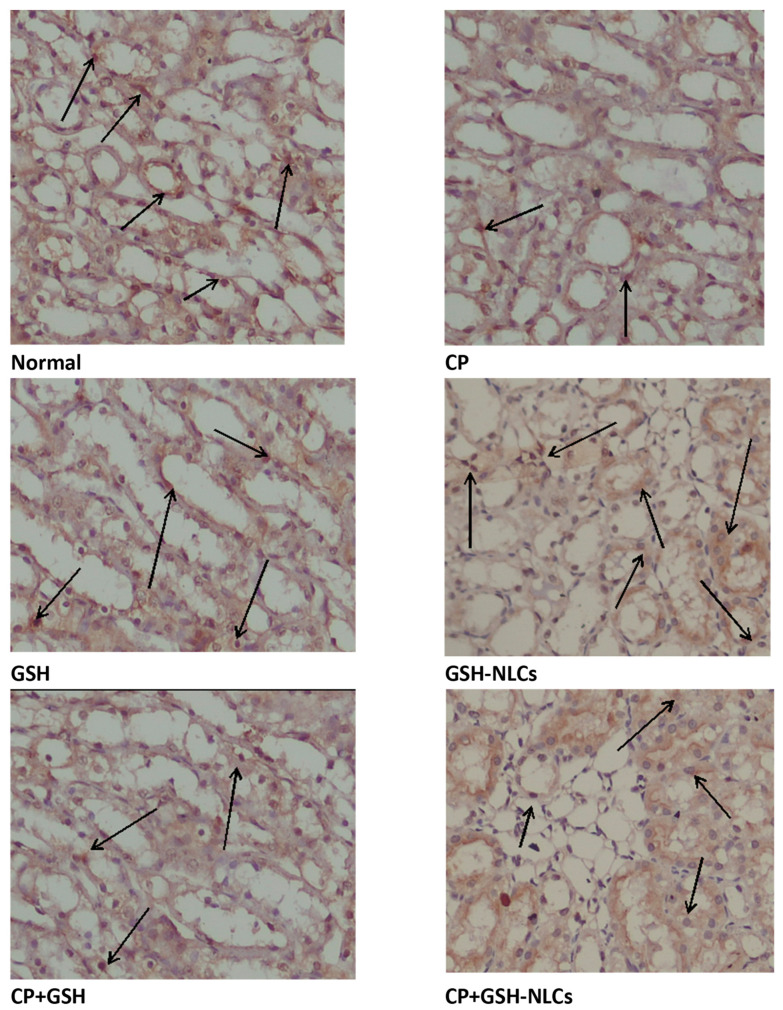
Immunohistochemistry results of Bcl-2 on renal tissue of **Normal** = Normal rats; **CP** = Cyclophosphamide-injured rats; **GSH** = glutathione-treated rats, **GSH-NLCs** = nano-glutathione-treated rats; **CP+GSH** = Cyclophosphamide + Glutathione-treated rats; **CP+GSH-NLCs** = Cyclophosphamide + nano-glutathione-treated rats (magnification ×200).

The renal tissue’s immunostaining revealed a normal distribution of Bcl-2 in the untreated normal group of animals, while remarkable reductions in Bcl-2 distributions and concentrations in renal tissue were observed in the CP-injured group. On the other hand, GSH-NSC oral administration significantly ameliorated the CP-depleting effects on Bcl-2, and the Bcl-2 concentration and distributions were found to be significantly higher than the CP-administered group. However, GSH administration could not produce any significant ameliorating effects on the CP-administered group and no ameliorating effects were observed (Figure 7).

### 2.12. Effects on NF-KB

**Figure 8:**
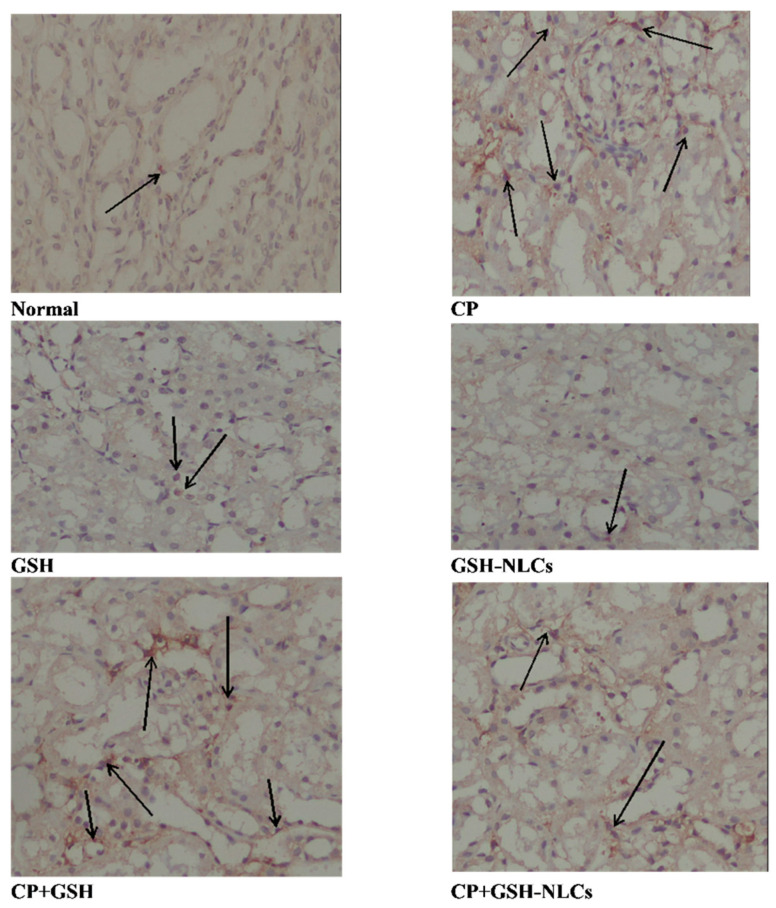
Immunohistochemistry results of NF-Kβ on renal tissue of **Normal** =Normal rats; **CP** = Cyclophosphamide-injured rats; **GSH** = glutathione-treated rats, **GSH-NLCs** = nano-glutathione-treated rats; **CP+GSH** = Cyclophosphamide + Glutathione-treated rats; **CP+GSH-NLCs** = Cyclophosphamide + nano-glutathione-treated rats (magnification ×200).

The renal tissue immunostaining for the detection of NF-Kβ revealed that CP i.p. administration produced a significant elevation in the NF-KB concentration compared to the normal group of animals. The results also revealed that GSH oral administration could not produce a remarkable inhibitory effect on the CP-induced upregulation of NF-KB. However, GSH-NLC oral administration, when used in combination with CP, significantly prevented CP’s upregulatory effect on NF-KB as shown in Figure 8.

## 3. Discussion

GSH-NLCs were successfully prepared using the emulsification-solvent evaporation technique followed by ultrasonication. In this formulation, a mixture of solid and liquid lipids was used as a lipid core of NLCs. The role of the liquid lipid was to provide lower crystalline conditions and higher amorphous matrix, which increases the NLCs’ capacity to entrap more drug molecules compared to other lipid nano-formulations. According to preliminary studies, using 1:1 weight ratios of stearic acid (solid lipid 300 mg) and oleic acid (liquid lipid 300 mg) provided GSH a higher solubility and encapsulation in the lipid mixture (Table 1) The presence of a high amount of solid lipid (stearic acid) enhanced the stability of the developed formulation. Further, the incorporation of a high content of oleic acid (liquid lipid) may lead to a large enough space to encapsulate drug molecules, thus, leading to improved encapsulation and loading efficiency. The presence of surfactant (Tween^®^ 80) effectively participated in the stability of the oily nano-droplets and emulsification of the aqueous and lipid phases during the solvent evaporation process of the preparation of the GSH-NLCs formulation. The selection of high concentrations of Tween^®^ 80 (2%), as hydrophilic surfactant, was performed to obtain a stable formulation. Based on the preliminary studies of GSH-NLCs, the formulation F7 was selected for further investigations. According to the results of the particle size and zeta potential (Table 2), the developed GSH-NLC (F7) formulation showed comparatively smaller particle sizes and a high negative zeta potential value, which indicated the stability of the fabricated colloidal dispersion. This value of zeta potential (−38 mV) indicated adequate physical stability of the nanostructured lipid carriers. The values were also consistent with the reported negative values of the zeta potential for lipid nano-formulations [32], providing equi-levels of the lipid carriers’ characteristics and properties of size, stability, and zeta potential.

The GSH-NLCs (F7) formulation exhibited high encapsulation efficiency (EE%) as shown in Table 1 and Table 2**,** which was attributed to the integration of oleic acid as liquid lipid into the solid lipid, stearic acid, whereby higher amounts of GSH were encapsulated into the more amorphous matrix domain [33,34]. The formulation was found in the nanosized range with a low value of PDI. The value of the polydispersity index (PDI) ≤ 0.5 is considered appropriate in drug delivery applications by representing a relatively homogenous distribution of NLCs [35]. Thus, use of the prepared nanostructured lipid carriers, as GSH-NLCs, provided a promising oral delivery system for glutathione (GSH) safe, protected, and stable oral delivery. This is because of the small particle sizes, which presented high affinity of the GSH-NLCs formulation to tissues, which was demonstrated in the treated animal group in comparison to the CP-administered group.

The biphasic release patterns of the GSH-NLC formulation may be due to the partitioning of the drug between the aqueous and lipid phases during the NLCs’ preparation. The drug on the outer oleic acid layer contributes to its initial burst release, and then is followed by sustained release of the drug from the inner lipid matrix. These results were consistent with previous literature works, which explained the biphasic drug release pattern for the NLC encapsulated drug. The controlled release of the drug from the loaded NLCs is predicted to improve drug residences from the NLCs within the tissue, thus improves tissue affinity for the drug, and enhancing its bioavailability through the oral route.

FT-IR studies were conducted to identify any chemical or physical interactions between the GSH and the other constituents of encapsulation. The FT-IR spectra (Figure 3) confirmed the successful formulation of glutathione as NLCs, as the characteristic peaks of GSH disappeared in the spectrum of GSH-NLCs, which confirmed the encapsulation of GSH into the lipid matrix.

The prepared GSH-NLCs were stable as a nano-formulation with the obtained particle size and PDI values. The presence of surfactant at a high concentration provided the physical and chemical stability of the prepared NLCs according to the stability data analysis (Table 3).

The renal contents of GSH in all animal groups were measured by the MLC technique. The results demonstrated that the i.p. administration of CP significantly decreased the renal tissue contents of GSH (0.41 ± 0.04 mmol/mg protein) in comparison to its contents in the untreated normal group of animals (0.73 ± 0.09 mmol/mg protein). The oral administration of pure free GSH has mild restoring effects on the renal GSH contents (0.48 ± 0.01 mmol/mg protein). However, oral administration of GSH-NLCs significantly protected the renal tissues against the CP-depleting effect of GSH, and an increase in the GSH levels in the renal tissue (1.41 ± −0.11 mmol/mg protein) over its levels in the untreated animals was observed.

The microscopic examinations of the renal tissue revealed that the CP administration induced tissue degeneration in renal tubules at the cortex, which is associated with endothelial cells’ swelling and vacuolization as well as multiple focal fibrosis, which could be a result of severe oxidative stress and ROS (reactive oxygen species) generation that was produced by the CP administrations [36,37]. The renal tissue-damaging effects of CP are attributable to its metabolite, acrolein, and its glutathione adduct, glutathione-propionaldehyde, produced in the presence of biocatalyst xanthine oxidase, O_2_^−^, and enzyme aldehyde dehydrogenase, which were able to generate excessive amounts of HO-, which is a highly reactive compound, and is mainly considered responsible for oxidative stress generation inside the cells, and thought to be producing large amounts of lipid peroxides [38,39]. Interestingly, the GSH-NLCs, when co-administrated with CP, remarkably protected the renal tissue from CP’s damaging effects (Figure 5). This could be partially attributed to the powerful anti-inflammatory and antioxidant properties of the glutathione molecule [40].

The present study also examined both the GSH and GSH-NLC oral administrations’ protective effects against CP-induced oxidative stress. The results showed that the concentrations of MDA, the main product of the lipid peroxidation, were remarkably increased inside the renal tissue by CP administration, which was also reported in a previous study [41]. The results confirmed that the GSH administration did not affect CP oxidative effects with elevated levels of MDA, while the GSH-NLCs administration significantly decreased the MDA concentration (Figure 6). These findings are an indicator of the enhanced oral bioavailability of GSH through its encapsulation as the NLC formulation, and subsequently the GSH-NLCs increased the renal tissue contents of GSH. Thereby, the oxidative stress and lipid peroxidation affected by CP were remarkably reduced through administration of the GSH-NLCs. Nonetheless, different studies have reported that CP administration can produce remarkable depletion of antioxidant enzymes, including GSH-Px, catalase, and SOD, which allows ROS accumulation and renal tissue damage [42,43]. In the present study, SOD was significantly decreased in the CP-administered animals group compared to the normal group of animals. On the other hand, GSH-NLCs noticeably ameliorated the effects of CP and kept the SOD concentrations close to its normal level. However, this effect was not observed through oral administration of pure free GSH when it was co-administrated with CP (Figure 6).

The cytokines, including IL-1β and NF-KB, are involved in the pathophysiology of renal injury. Elevated levels of these cytokines were observed in CP-based treatment, and their attenuation has the potential to ameliorate renal injury [44,45]. It also a known fact that GSH is one of the most potent antioxidants and has demonstrated anti-inflammatory properties together with modulation of many inflammatory markers to control inflammation [46]. In the present study, the CP group showed elevated levels of the IL-1β serum concentration and upregulation of the NF-KB (Figure 6 and Figure 8) concentration inside renal tissue at similar levels to the reported data, as compared to the normal group of animals [47,48]. The elevated levels of the cytokines, i.e., IL-1β and NF-KB, were remarkably improved by GSH-NLC administration, while these effects were not significantly affected by the GSH oral administration. Again, these results of the IL-1β and NF-KB levels in the animal groups are confirmatory of the anti-inflammatory and nephron-protective activity of the prepared nanoformulation, GSH-NLCs.

Urea and creatinine determinants are well-established biochemical analysis parameters commonly used to assess and evaluate renal function. They are reflected in the renal glomerular filtration rate (GFR), and their concentrations reflect the severity of renal injury [49]. A substantial increase in the urea and creatinine concentrations was observed in the present study in the CP-administered animal group, which was in agreement with previous studies [18,50]. Nevertheless, GSH-NLCs’ co-administration with CP inhibited the urea and creatinine increment as normally produced by CP, and this provided clear evidence of the GSH-NLCs’ reno-protective efficacy. Furthermore, Bcl-2 plays a crucial role in the regulation of renal cell apoptosis, and different studies have demonstrated that Bcl-2 was upregulated when renal tissues were exposed to moderate injury [51,52]. However, in case of severe distress conditions, such as high oxidative stress, Bcl-2 concentrations were dramatically decreased compared to normal levels [53,54]. This was in agreement with our findings. On the other hand, the present study also showed that GSH-NLCs’ oral administration protected Bcl-2 protein from depletion as shown by immune-staining experimental observation (Figure 7). This could be attributed to the antioxidant properties of the GSH, outreached to the renal tissue as the GSH-NLCs formulation. However, the pure free GSH administration could not preserve the Bcl-2 concentration when co-administered with CP.

This study provided the application’s advantages of the GSH-NLCs when co-administered with CP in animal models. On a more elaborate front, the study needs to be extended to fully blown cancer models, and modulated with other anticancer agents and agents that produce ROS-based damage, and removal of oxidative stress in different diseases. GSH has potential as an antioxidant agent, which could be utilized as GSH-NLCs in different biological and physiological conditions. GSH is a biomolecule that is safe, biocompatible, and needs the lowest quantities of the compound in comparison to other synthetic and plant-based antioxidants currently in use.

## 4. Material and Methods

### 4.1. Chemicals and Reagents

Glutathione (GSH, reduced) was obtained from Sigma Chemicals (St. Louis, MO, USA). Tween^®^ 80 and oleic acid were purchased from Alpha Chemicals Co., Cairo, Egypt. HPLC-grade chloroform, methanol, and acetonitrile, and analytical-grade sodium acetate, disodium hydrogen phosphate, and sodium lauryl sulfate were purchased from Merck, Darmstadt, Germany. Cyclophosphamide and reduced glutathione were purchased from Sigma Chemicals, St. Louis, MO, USA. Ultra-pure water was prepared by using a Milli Q Plus water purification system (Millipore, Milford, MA, USA). The standard solution of GSH was prepared by dissolving 50 mg/L of GSH in 10 mM EDTA to prevent oxidation by trace metals. The standard working solution was prepared through dilution with the HPLC mobile phase.

### 4.2. Preparation of Glutathione-Loaded Nanostructured Lipid Carriers (GSH-NLCs)

The emulsification-solvent evaporation technique was used to load GSH within the NLCs. The method was followed by ultrasonication as reported earlier with slight modifications [27]. In brief, the lipid phase was composed of stearic acid (solid lipid, 300 mg) and oleic acid (liquid lipid, 300 mg) as well as GSH dissolved in ethanol (2 mL, 1:1, *v*/*v*) at 70 °C. In total, 20 mL of the distilled water containing 2% of the Tween^®^ 80 were heated at 70 °C to prepare the aqueous phase. Then, both phases, aqueous and lipid phases, were mixed at the same temperature (70 °C) using 2000 rpm stirring for 15 min. The resulting pre-emulsion obtained from the mixture of the aqueous and lipid phases was sonicated by a probe-type sonicator (Cole-Parmer, Vernon Hills, IL, USA) for 10 min at pulse-ON for 3 s and pulse-OFF for 5 s (40 W). The obtained dispersion was allowed to cool to RT under continuous stirring for 60 min at 1000 rpm for 1 h to obtain the GSH-NLCs dispersions. The effects of the oleic acid to stearic acid ratio on the encapsulation efficiency of the formulations were evaluated. Plain NLCs were fabricated as a control free of GSH. Seven different formulations with different compositions of liquid and solid lipid were prepared. Table 1 illustrates the composition of the fabricated glutathione-loaded nanostructured lipid carriers.

### 4.3. RP-HPLC: Analytical Procedure

The HPLC system consisted of a binary solvent pump (Agilent 1260 Infinity, Agilent Technologies, Waldbronn, Germany), thermostat-controlled column compartment, autosampler, and diode array detector (Agilent 1260 Infinity). The output signals were monitored and processed using OpenLAB CDS ChemStation software. All solutions were degassed by ultra-sonication (Power Sonic 420, Labtech, Seoul, Korea) and filtered through a 0.45-µm Nylon filter (PALL life sciences, Alexandria, VA, USA).

A validated micellar liquid HPLC method for quantification of GSH was developed. A reversed phase C18 column (Pursuit-3 C18, 150 mm × 4.6 mm internal diameter, and 3 μm particle size, Agilent Technologies, Middelburg, Netherland) was used as the stationary phase, while the mobile phase consisted of 95% potassium phosphate buffer (20 mM, pH 2.5) containing 0.15 M sodium lauryl sulphate (SDS) and 5% acetonitrile. The chromatographic run was carried out at 30 °C, in isocratic mode with a mobile phase flow rate of 0.7 mL/min, while the injection volume and the detection wavelength were 20 µL and 210 nm, respectively.

### 4.4. Characterization of Glutathione-Loaded Nanostructured Lipid Carriers (GSH-NLCs)

#### 4.4.1. Encapsulation Efficiency and Loading Capacity

The encapsulation (EE%) of GSH within different formulations of NLCs was estimated indirectly by measuring free unloaded GSH by the proposed HPLC method, after separation via cooling centrifugation at 4 °C, 14,000 rpm, for 60 min, using a bench-top refrigerated centrifuge (Centurion Scientific Ltd., West Sussex, UK) [3]. EE (%) was calculated according to the following equation:

EE (%) = ((T − C))/T × 100 (1)

where T is the total amount of GSH and C is the amount of free unentrapped extract in the supernatant. Each experiment was performed in triplicate.

On the other hand, the solution of GSH-loaded nanostructured lipid carriers was dried and weighed. The loading capacity (DL%) of the different GSH nanostructured lipid carrier formulations was determined according to the following equation:

DL (%) = ((T − C))/(Total material weight) × 100 (2)

#### 4.4.2. Particle Size, and Zeta Potential Measurements

The particle size distribution and poly dispersity index (PDI) of the fabricated GSH-NLCs were measured at 25 °C by the dynamic laser light scattering (DLS) technique using the Zetasizer Nano ZS (Malvern Instruments, Worcestershire, UK) equipped with a backscattered light detector operating at 173°. The zeta potential of the NLCs dispersion was determined by laser Doppler anemometry using a Malvern Zetasizer Nanoseries ZS. The measurements were performed in triplicate.

#### 4.4.3. Particles Morphology

The surface morphology of GSH-NLCs was visualized using scanning electron microscopy (SEM) (Jeol, JSM-5200, Tokyo, Japan). The sample of GSH-NLCs was prepared by placing a droplet onto an aluminum specimen stub, dried overnight, and sputter-coated with gold prior to imaging. An acceleration voltage of 15 kV was utilized for SEM.

#### 4.4.4. In Vitro Drug Release Studies

The in vitro release of the GSH-NLC formulation compared with the pure GSH solution was analyzed using the dialysis membrane diffusion technique. Phosphate buffer saline (PBS) pH 7.4 was selected as the release vehicle. In total, 1 mL of the GSH-NLCs formulation, equivalent to 50 mg GSH, was put over a previously soaked cellulose membrane (Spectro/Pore membranes, molecular weight cut-off 12–14 kDa)) fitted at the lower end of a glass cylinder. The glass cylinder was then dipped in a beaker containing a PBS system at 37 ± 0.5 °C and agitated at a fixed speed of 100 rpm using a thermostatically controlled water bath (Gesellschaft Labor Technik M.B.H. & Co., GFL., Gesellschaft, Germany). Aliquots (5 mL) were removed and substituted with a freshly prepared buffer medium. The drug content was estimated via the MLC (micellar liquid chromatography) method at predefined time points for 24 h. The in vitro release experiment was repeated in triplicate. Different mathematical models were applied, in order to measure the kinetics and mechanism of drug release from the prepared NLCs formulation.

#### 4.4.5. Fourier Transform Infrared (FT-IR) Spectroscopy

The infrared spectra of glutathione and the physical mixture of drug with stearic acid, oleic acid, and glutathione nanostructured lipid carrier (GSH-NLC) were evaluated using a Nicolet 6700 FT-IR spectrometer (Thermo Fisher Scientific, Waltham, MA, USA). All samples were mixed with potassium bromide (KBr) of spectroscopic grade and compressed into disks using a hydraulic press (15,000 Ib). Samples were scanned from 4000 to 400 cm^−1^.

#### 4.4.6. Stability Studies

The stability of the developed formulation was determined by storing it at 4 °C and room temperature in a sealed 20 mL glass vial. The size, PDI, and zeta potential values were recorded at predefined time intervals (fresh preparation, and 4 weeks after formulation and storage).

### 4.5. Animal Study

In the present study, 48 male Sprague dewily rats weighing 200–220 g were used. Rats were purchased from Biological Products & Vaccines Holding Company, Helwan Farm, Cairo, Egypt. Rats were placed in a standard cages at a fixed temperature (23  ±  1 °C) and humidity, with a 12 h light/12 h dark cycle. Food and water were provided ad libitum. The animals were kept freely one week before the experimental process to acclimatize to their new environment. This study was designed and conducted according to ethical norms approved by the ethical committee at the faculty of Pharmacy, South Valley University (Approval P1004).

#### 4.5.1. Experimental Designs

The 48 animals were randomly divided into six groups of (*n* = 8) as follows:

Group 1: animals were injected with normal saline (2.5 mL/kg, i.p.) on days 3, 4, 5, 19, 20, and 21 and given daily oral normal saline 3 mL from day 1 to day 22; Group 2: animals were i.p. injected with 75 mg/kg of CP on days 3, 4, 5, 19, 20, and 21 as single injections to induce nephrotoxicity [36]; Group 3: animals were administrated 3 mL of 100 mg/kg GSH (dissolved in normal saline) as a daily dose; Group 4: animals were administrated 3 mL of 100 mg/kg GSH-NLCs (dissolved in normal saline) as a daily dose; Group 5: animals were i.p. injected with 75mg/kg of CP on days 3, 4, 5, 19, 20, and 21 + daily dose of GSH (100 mg/kg); Group 6: animals were i.p. injected with 75mg/kg of CP on days 3, 4, 5, 19, 20, and 21 + daily dose of GSH-NLCs (100 mg/kg). After 24 h from the last administered dose and under light ether anesthesia, blood samples were collected from the heart (inferior vena cava) in clean dry test tubes and centrifuged at 3000 rpm for 10 min and serum was separated and refrigerated at −20 °C for further analysis. One kidney from each animal was immediately dissected out, washed with ice-cold saline, and homogenized using 100 mmol KH2PO4 buffer containing 1 mmol EDTA (pH 7.4) and centrifuged at 12,000× *g* for 30 min at 4 °C, resulting in 10% homogenate. The supernatant was collected and stored at −80 °C for further analysis. The other kidneys were preserved in 10 % formalin for further histopathological and immunohistochemical examination.

#### 4.5.2. Histopathological and Immunohistochemical Examinations

Kidney samples were embedded in paraffin wax and cut into 5 μm sections then stained with hematoxylin and eosin and periodic acid Schiff’s stain. Stained tissues were examined under a light microscope. On the other hand, renal tissue samples (5 µm) were de-paraffinized and examined for the presence of NF-KB and Bcl-2 using a standard immunohistochemical procedure. Sample analyses were performed in the Pathology Department at the Faculty of Medicine, South Valley University using an Olympus microscope with 200× magnification.

##### Determination of Total Proteins

The total protein concentration inside renal tissue homogenates was determined using the Bradford technique.

##### Determination of Renal Function Tests

Creatinine and urea serum concentrations were determined as markers of kidney function, using commercial kits from Biodiagnostic, Cairo, Egypt.

##### Determination of Nitric Oxide Concentrations (NO)

The levels of nitric oxide inside renal tissue were determined using a commercial kit from (Biodiagnostics, Cairo, Egypt) and following the manufacturer’s instructions.

##### Assessments of Superoxide Dismutase (SOD)

The activity of SOD inside renal tissue was assessed using a standard kit (Biodiagnostics, Cairo, Egypt) according to the manufacturer’s protocol. Hence, serial dilutions of standard SOD and samples were added to each well, followed by radical detector and xanthine oxidase. The plate was shaken and incubated at room temperature for 30 min. Absorbance was read at 440–460 nm using the ELISA microplate reader.

##### Assessment of Inflammatory Marker (IL-Iβ)

IL-1β serum concentrations were determined using the corresponding rat-specific ELISA kit (Sigma-Aldrich, St. Louis, MO, USA) and following the manufacturer’s protocols.

##### Malondialdehyde (MDA) Determination

The MDA levels inside renal tissue homogenate were determined using the spectrophotometric method based on the reaction between the product of lipid peroxidation (MDA) and thiobarbituric acid, producing a pink color, with its absorbance measured at 532 nm.

### 4.6. Statistical Analyses

Statistical analysis using graph-pad prism version 9.2.0 (332) was performed and values were presented as means ± standard error of the means (SE), *n* = 8. Comparison between groups was performed using one-way analysis of variance followed by the Tukey–Kramer test and the difference was considered significant when *p* < 0.05.

## 5. Conclusions

Stable oral formulation of GSH was prepared through drug encapsulation as nanostructured lipid carriers. The formulation, glutathione-nanolipid carriers (GSH-NLCs), was prepared to avoid intestinal enzymes’ breakdown and structural alterations of GSH in an oral delivery module. The prepared nano-formulation was orally delivered, and was found to be safe, stable, biocompatible, and provided required bioavailability concentrations of the chemically and physico-chemically unaltered GSH, which was confirmed by the significant presence of GSH in the renal tissue. GSH-NLCs ameliorated the nephrotoxic effects induced by cyclophosphamide, a frequently used anticancer agent, which has potential for extended and enhanced use of cyclophosphamide in the treatment of different cancer types. The cyclophosphamide-based toxicity and adverse effects were successfully controlled through the GSH supplication. The drawbacks of the pure free glutathione as being chemically altered, and specifically being oxidized, were eliminated by the currently prepared nano-formulation. Clinical applications for the mixture, cyclophosphamide/GSH-NLCs, in the treatment of various cancers are recommended to pass-on its effectiveness and evaluate its side effects, especially for the kidneys.

## Figures and Tables

**Table 1:** Composition of glutathione-loaded nanostructure lipid carrier.

Formulation	Oleic Acid (mg)	Stearic Acid (mg)	Tween^®^ 80 (mg)	EE%	LD%
F1	100	200	200	52 ± 1.4	1.5 ± 0.08
F2	200	100	200	55 ± 1.3	2.3 ± 0.05
F3	100	100	200	49 ± 0.9	3.2 ± 0.09
F4	200	200	200	59 ± 1.2	4.1 ± 0.03
F5	300	100	200	62 ± 0.87	5.7 ± 1.1
F6	100	300	200	65 ± 1.5	5.9 ± 0.9
F7	300	300	200	79.8 ± 1.9	6.78 ± 0.05

**Table 2:** Characterization of the selected GSH-Loaded Nanostructured Lipid Carriers (GSH-NLCs).

Characterization Property	Value
Particle size, nm	452.4 ± 33.19
Polydispersity index	0.500 ± 0.12
Zeta potential, mV	−38.5 ± 1.4
Encapsulation efficiency, %	79.8 ± 1.9
Loading capacity, %	6.78 ± 0.05

**Table 3:** Vesicle size, zeta potential, polydispersity index (PDI), and encapsulation efficiency (EE %) GSH-loaded NLCs stored at 4 °C and at room temperature for one month.

Storage Temperature	4 °C	At Room Temperature (25 °C)
Size (nm)	580.2 ± 10.25	598.9 ± 12.12
PDI	0.519 ± 0.14	0.580 ± 0.08
Zeta potential (mV)	−32.6 ± 0.09	−30.6 ± 0.34
EE%	75.87 ± 1.3	73.2 ± 1.5

**Table 4:** Statistical parameters for the individual calibration curve.

Parameter	Value
λ_max_	210 nm
Linearity range (µgmL^−1^)	5.0–25.0
LOD (µgmL^−1^)	0.439
LOQ (µgmL^−1^)	1.33
R^2^	0.9998
Regression Equation	A* = bx** + *a*
Slope (×10^6^) ± SD	585.03 ± 0.022
Intercept (×10^6^) ± SD	77.83 ± 0.047

A* is peak area. x** is concentration in µgmL^−1^.

**Table 5:** System Suitability Parameters for the Determination of GSH by the HPLC Method.

HPLC Parameter	GSH	Acceptable Limits
Asymmetry factor	0.933	>1.5
Theoretical plates/m	3911	<200
Tailing factor	1.23	>2.0
HETP (cm)	0.0038	

## Data Availability

All the data are represented in the manuscript and Supplementary File.

## Supplementary Materials

The following are available online, Table S1: Intra-Day and Inter-Day Accuracy and Precision for the Determination of GSH by the Proposed HPLC Procedure, Figure S1: Scheme of GSH-NLCs preparation.

## Sample Availability

Samples of the GSH-NLCs are available from the authors.
