# Supplementary material for: Nano-Structured Lipid Carrier-Based Oral Glutathione Formulation Mediates Renoprotection against Cyclophosphamide-Induced Nephrotoxicity, and Improves Oral Bioavailability of Glutathione Confirmed through RP-HPLC Micellar Liquid Chromatography
Source: Molecules. 2021 Dec 10;26(24):7491. doi: 10.3390/molecules26247491 (PMC8706828; doi:10.3390/molecules26247491)
Supplement: Supplementary file 1 [file molecules-26-07491-s001.zip › molecules-1489262-supplementary.pdf]

Table S1: Intra-Day and Inter-Day Accuracy and Precision for the Determination of GSH by the Proposed HPLC Procedure:

**Analyte**	**Injected (µg/mL)**	**Intra-day**			**Inter-day**		
		**Found*** **(Conc.± SD)**	**Accuracy (R %)**	**Precision (RSD %)**	**Found*** **(Conc.± SD)**	**Accuracy (R %)**	**Precision (RSD %)**
**GSH**	5.0	4.96±0.04	99.49	0.94	4.98±0.02	99.79	0.04
	17.0	17.02±0.14	99.23	0.85	16.89±0.11	99.39	0.71
	25.0	25.09±0.36	100.62	1.44	24.97±0.35	99.88	0.14

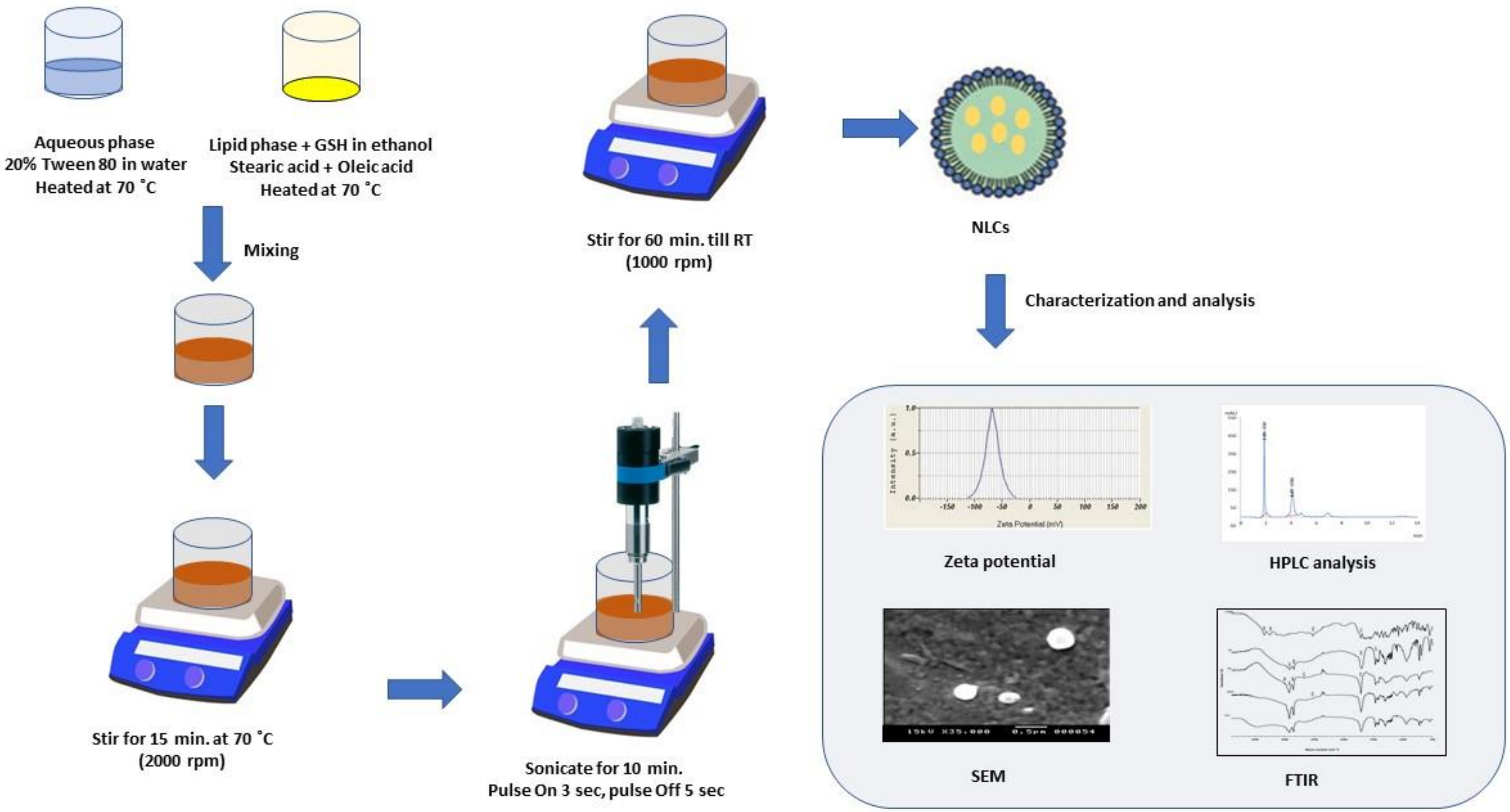

Figure S1: Scheme of GSH-NLCs preparation.
